# Dental Caries Experience (DMFT) and Oral Hygiene Status Among 18-Year-Old Adolescents in Two Bulgarian Municipalities: A Cross-Sectional Study

**DOI:** 10.3390/children13081087

**Published:** 2026-08-16

**Authors:** Veselina Kondeva, Velina Stoeva, Sevda Rimalovska, Rumyana Stoyanova

**Affiliations:** 1 Department of Pediatric Dentistry, Faculty of Dental Medicine, Medical University of Plovdiv, 4002 Plovdiv, Bulgaria; veselina.kondeva@mu-plovdiv.bg; 2 Department of Epidemiology and Disaster Medicine, Faculty of Public Health, Medical University of Plovdiv, 4002 Plovdiv, Bulgaria; velina.stoeva@mu-plovdiv.bg; 3 Department of Health Management and Health Economics, Faculty of Public Health, Medical University of Plovdiv, 4002 Plovdiv, Bulgaria

**Keywords:** DMFT index, oral hygiene index, prevalence of dental caries, filled teeth, missing teeth

## Abstract

**Highlights:**

**What are the main findings?**

- Untreated dental caries was prevalent among 18-year-old students, with a mean DMFT of 7.48 ± 4.08.
- Male sex was significantly associated with poorer oral hygiene, while rural participants had significantly higher DMFT values.

**What are the implication of the main finding?**

- The findings highlight the need for targeted oral-health promotion and preventive strategies among adolescents across different residential and sex groups, with particular attention to those with poorer oral health indicators.
- Combining current oral hygiene assessment with cumulative caries experience may help identify groups requiring enhanced preventive attention.

**Abstract:**

**Background**: As a highly prevalent chronic non-communicable disease in children and teens, dental caries continues to be a major global public health issue. Effective prevention and planning rely heavily on the systematic monitoring of oral health at key developmental ages. **Objectives**: This study aimed to assess dental caries prevalence and experience, alongside oral hygiene levels, among 18-year-olds using the DMFT and OHI-S indices. Additionally, differences in caries experience and oral hygiene status according to sex and place of residence were examined. **Methods**: A cross-sectional study was conducted (May–June 2026) involving 480 18-year-old adolescents. A stratified quota sampling design ensured equal representation by sex (male/female) and residence (urban/rural). Following informed consent, clinical indices were recorded and statistically analyzed. **Results**: Participant distribution by sex and residence was balanced. For the overall cohort, the mean DMFT index was 7.48 ± 4.08 (decayed: 3.91 ± 3.59; missing: 0.13 ± 0.43; filled: 3.43 ± 3.33). Females exhibited a significantly higher number of filled teeth (*p* = 0.004). Urban–rural comparisons revealed significant disparities in decayed (*p* = 0.001) and missing teeth (*p* = 0.015), as well as the total DMFT index (*p* = 0.002). The mean OHI-S score was 1.89 ± 1.01. Sex-based analysis revealed significantly poorer oral hygiene in males across all parameters: DI-S (*p* = 0.034), CI-S (*p* = 0.004), and OHI-S (*p* = 0.006). No significant differences in OHI-S components were observed based on residence. **Conclusions**: Residence was significantly associated with caries experience, while females had significantly more filled teeth than males. Poorer oral hygiene was associated with higher DMFT scores. These findings support consideration of targeted preventive oral health strategies for population groups with poorer oral health indicators and may provide preliminary evidence relevant to regional oral health surveillance and preventive planning.

## 1. Introduction

Oral diseases present a substantial public health challenge during childhood and adolescence. The World Health Organization (WHO) estimates that these conditions affect over 3.5 billion people globally, with untreated dental caries being the most prevalent. In children, these pathologies severely compromise quality of life, physical growth, nutrition, and systemic health [1].

The 2021 World Health Assembly Resolution on Oral Health marked a paradigm shift toward preventive care and the inclusion of dentistry within national universal health coverage frameworks. Aligning with this framework, the WHO’s 2023–2030 Action Plan targets a 25% reduction in untreated dental caries by 2030 [1]. Similarly, the World Dental Federation’s (FDI) Vision 2030 emphasizes intersectoral collaboration to integrate oral health services into primary healthcare systems [2].

As a highly prevalent chronic non-communicable disease with a multifactorial microbial etiology, dental caries persists as a major global public health challenge in children and adolescents [3,4]. The disease arises from oral microbiome dysbiosis, which is accelerated by frequent fermentable carbohydrate intake, poor oral hygiene, and extended exposure times [5].

Extensive literature confirms that dental caries is the most prevalent pediatric pathology, highlighting the critical need for systematic, continuous monitoring of oral health across different age groups [6]. One of the most widely used epidemiological indicators for assessing the prevalence and severity of dental caries is the DMFT/dmft index (Decayed, Missing, and Filled Teeth). This index quantifies the number of decayed teeth (D/d), teeth missing due to caries (M/m), and filled teeth (F/f), providing an objective measure of caries experience within a population [7].

Assessing the DMFT/dmft index across distinct dental development stages enables researchers to evaluate caries prevalence and severity, while simultaneously identifying risk factors associated with diet, oral hygiene behaviors, socioeconomic circumstances, and healthcare accessibility [8]. Consequently, such epidemiological data are vital for designing targeted preventive programs and strategic initiatives to enhance oral health outcomes in pediatric and adolescent populations [9,10].

Sociodemographic disparities heavily influence oral disease distribution, which is markedly skewed toward underserved and marginalized populations. Susceptibility to severe oral health conditions is strongly associated with socioeconomic determinants, including income, occupation, and education. This persistent trend initiates during infancy, spans into older age, and is globally reproducible across varying national economies.

Adolescence is delineated by the World Health Organization as the developmental period from 10 to 19 years of age [11]. Concurrently, the American Academy of Pediatrics categorizes this developmental stage into three distinct phases: early (11–14 years), middle (15–17 years), and late adolescence (18–21 years) [12]. Importantly, late adolescence marks a transitional period where lifestyle factors—such as dietary choices, oral hygiene routines, personal identity, and health motivations—are consolidated, laying the foundation for adult oral health behaviors [13].

In Bulgaria, there is a scarcity of recent epidemiological data regarding the oral health status of late adolescents. This gap in the national literature provides the rationale for the present study, which evaluates dental caries experience and oral hygiene levels among 18-year-olds across different settlement types in two Bulgarian municipalities.

### 1.1. Objective

The primary objective of this study was to evaluate dental caries experience and oral hygiene status among 18-year-old adolescents using the DMFT and OHI-S indices. Secondary aims included conducting a comparative analysis across sex and residential demographics (urban vs. rural).

### 1.2. Working Hypothesis (H_1_)

It is hypothesized that dental caries experience and oral hygiene levels differ significantly according to sex and place of residence (urban versus rural) among 18-year-olds.

### 1.3. Null Hypothesis (H_0_)

It is hypothesized that there are no statistically significant differences in dental caries experience (DMFT index) or oral hygiene levels (OHI-S index) among 18-year-olds according to sex and place of residence (urban versus rural).

## 2. Materials and Methods

This cross-sectional study was conducted between May and June 2026 among 480 18-year-old adolescents from two municipalities within the same Bulgarian district, including one urban center and two nearby rural communities with similar geographical and climatic characteristics. Within this geographical area, two rural communities were selected at random, with the additional criterion that they were located in proximity to the major urban center in order to minimize differences in geographical and climatic conditions. Each rural community had a single school, and these schools constituted rural recruitment sites. In the major urban center, two schools were also selected at random.

All 18-year-old students in the selected schools were informed about the study and invited to participate. Participation was voluntary, and only students who expressed willingness to participate and provided written informed consent were included. No formal records of students who declined participation were maintained, as no students who attended for the clinical examination subsequently formally declined participation. Consequently, exact refusal and participation rates could not be calculated.

A stratified quota sampling approach was used to ensure balanced representation by place of residence and sex. Given the relatively limited number of eligible adolescents in the rural communities, a quota of 120 males and 120 females was established for the rural group. The same quotas were applied to the urban group to ensure balanced comparisons between urban and rural participants, resulting in four equally sized groups: 120 urban males, 120 urban females, 120 rural males, and 120 rural females. Recruitment continued until the predefined quotas were reached. No formal a priori sample-size calculation was performed.

Clinical oral examinations were performed in school medical offices by three previously trained dentists, each with more than 15 years of professional experience. Examinations were conducted using disposable dental instruments and a portable light source. Prior to the main investigation, a pilot study involving 30 participants was conducted; these participants were not included in the final epidemiological analysis. The pilot study was performed to assess the feasibility of the study procedures and to standardize the clinical examination protocol.

Clinical data were documented using a customized statistical form specifically developed for this investigation. Prior to enrollment, written informed consent was secured from all participants following a detailed briefing regarding the study’s aims, objectives, and anticipated public health benefits. Individuals with systemic disease or those who declined to participate were excluded from the final cohort.

Dental caries was recorded using the DMFT index (Decayed, Missing, and Filled Teeth) according to the World Health Organization (WHO) diagnostic criteria (1997) [14]. These criteria were retained to ensure a standardized examination procedure and internal consistency across the predefined study groups. Oral hygiene status was evaluated using the Oral Hygiene Index–Simplified (OHI-S) developed by Greene and Vermillion (1964) [15].

The DMFT score for each participant was calculated as the sum of decayed (DT), missing due to caries (MT), and filled (FT) permanent teeth. The prevalence of untreated dental caries was defined as the proportion of participants with at least one untreated decayed tooth (D > 0), while caries experience was assessed using the DMFT index. Only clearly cavitated caries lesions were recorded, and the third molars were not included in the DMFT calculation. Radiographic assessment of dental status was not possible, which is acknowledged as a limitation of the study.

The Simplified Oral Hygiene Index (OHI-S) comprises two distinct clinical components: the Debris Index–Simplified (DI-S) and the Calculus Index–Simplified (CI-S), evaluated across six representative tooth surfaces: the buccal surfaces of the maxillary first molars (16, 26) and the maxillary right central incisor (11), alongside the lingual surfaces of the mandibular first molars (36, 46) and the mandibular left central incisor (31).

Debris (DI-S) and calculus (CI-S) accumulations were scored on a scale from 0 to 3 based on surface coverage. For the DI-S, scores were assigned as follows: 0 indicated no debris or extrinsic stain; 1 represented soft debris covering ≤ one-third of the surface or extrinsic stains without other debris; 2 signified debris covering between one-third and two-thirds; and 3 denoted coverage exceeding two-thirds of the tooth surface. Parallel criteria were applied to the CI-S, where a score of 0 indicated no calculus; 1 represented supragingival calculus covering ≤ one-third; 2 denoted supragingival calculus covering between one-third and two-thirds, or individual flecks of subgingival calculus; and 3 signified supragingival calculus exceeding two-thirds, or a continuous, heavy band of subgingival calculus. The DI-S and CI-S scores were calculated by summing the scores obtained for the examined tooth surfaces and dividing by the number of surfaces examined. The OHI-S score was calculated as the sum of the DI-S and CI-S scores.

The study procedures followed a structured sequence of clinical and analytical phases. Initially, a comprehensive clinical intraoral examination was performed on the selected adolescent population. Following these screenings, individual and cohort DMFT values were systematically calculated to evaluate the distribution of untreated decayed (DT), missing (MT), and filled (FT) permanent teeth, while oral hygiene status was concurrently quantified utilizing the OHI-S index. Subsequently, a comparative statistical analysis stratified by biological sex and geographic place of residence was conducted to identify variations between the demographic groups. Ultimately, these compiled metrics enabled a thorough epidemiological assessment of overall dental caries prevalence, caries experience, and oral hygiene levels across the entire study population.

### Statistical Analysis

Data were entered into Microsoft Excel 2010 and analyzed using the Statistical Package for the Social Sciences (SPSS), version 23.0. The distribution of continuous variables was assessed using the Kolmogorov–Smirnov test together with visual inspection of histograms and Q–Q plots. Although some variables deviated from a normal distribution, independent-samples *t*-tests were used because of the relatively large sample size (*n* = 480), for which parametric tests are considered robust to moderate violations of normality. As a sensitivity analysis, Mann–Whitney U tests were additionally performed and yielded conclusions consistent with those obtained from the independent-samples *t*-tests. Associations between categorical variables were examined using the chi-square test, and correlations were assessed using Spearman’s rank correlation coefficient. Statistical significance was set at *p* < 0.05.

The study was approved by the Research Ethics Committee at the Medical University of Plovdiv (Opinion P-KHE-69/8 May 2026).

## 3. Results

The study cohort comprised 480 participants with a balanced demographic distribution, consisting of 50% males (*n* = 240) and 50% females (*n* = 240), as well as an equal split between urban (50%, *n* = 240) and rural (50%, *n* = 240) residents.

The prevalence of untreated dental caries at the time of examination was 79.2% (*n* = 380), with a mean DMFT index of 7.48 ± 4.08. For the entire sample, the mean number of decayed teeth (DT) was 3.91 ± 3.59, the mean number of missing teeth (MT) was 0.13 ± 0.43, and the mean number of filled teeth (FT) was 3.43 ± 3.33.

Sex-stratified analysis of these DMFT components revealed variations between the groups; however, a statistically significant difference was isolated exclusively for filled teeth, which were markedly higher among females (*p* = 0.004). No statistically significant sex-based differences were detected for the decayed or missing components, or the cumulative DMFT index (*p* > 0.05) (Table 1).

Residential analysis of the DMFT index revealed marked geographic variations between urban and rural cohorts (Table 2). Statistically significant differences were identified in the number of untreated decayed teeth (*p* = 0.001), missing teeth (*p* = 0.015), and the cumulative DMFT index (*p* = 0.002), with markedly higher mean values consistently recorded among rural residents.

For the entire study population, the mean Simplified Oral Hygiene Index (OHI-S) was 1.89 ± 1.01, with mean values of 1.66 ± 0.78 for the Debris Index (DI-S) and 0.23 ± 0.38 for the Calculus Index (CI-S). Sex-stratified analysis revealed statistically significant differences across all examined oral hygiene parameters, with males consistently exhibiting higher scores than females (Table 3). Specifically, males demonstrated significantly higher accumulation for the DI-S (1.74 ± 0.84 vs. 1.59 ± 0.71; t = 2.122, *p* = 0.034), the CI-S (0.28 ± 0.44 vs. 0.18 ± 0.31; t = 2.922, *p* = 0.004), and the cumulative OHI-S score (2.02 ± 1.12 vs. 1.77 ± 0.87; t = 2.746, *p* = 0.006).

In contrast to the sex-based findings, a comparative evaluation based on the place of residence revealed no statistically significant differences across any of the examined oral hygiene parameters (Table 4). The distribution of scores remained highly comparable between the subgroups, with rural and urban adolescents displaying statistically equivalent mean values for the DI-S (1.68 ± 0.81 vs. 1.64 ± 0.76; t = −0.655, *p* = 0.513), the CI-S (0.25 ± 0.39 vs. 0.21 ± 0.38; t = −0.950, *p* = 0.342), and the cumulative OHI-S index (1.93 ± 1.06 vs. 1.85 ± 0.96; t = −0.865, *p* = 0.387).

Spearman’s correlation analysis demonstrated a significant positive association between oral hygiene status (OHI-S) and specific components of the DMFT index (Table 5). For male participants, higher OHI-S scores correlated moderately with a greater number of untreated decayed teeth (r_s_ = 0.341, *p* < 0.001) and elevated cumulative DMFT values (r_s_ = 0.347, *p* < 0.001). Among female adolescents, statistically significant but weaker positive correlations were similarly observed for decayed teeth (r_s_ = 0.252, *p* < 0.001) and the total DMFT score (r_s_ = 0.307, *p* < 0.001). Conversely, no statistically significant correlations were detected between OHI-S and either missing teeth or filled teeth in either sex (*p* > 0.05), with all coefficients remaining near zero.

Regarding the place of residence, Spearman’s correlation analysis also demonstrated significant positive associations between OHI-S scores and specific DMFT components (Table 6). Within the urban cohort, higher OHI-S scores correlated weakly but significantly with untreated decayed teeth (r_s_ = 0.194, *p* = 0.003) and the total DMFT index (r_s_ = 0.264, *p* < 0.001). Among rural participants, statistically significant positive correlations were observed for decayed teeth (r_s_ = 0.388, *p* < 0.001) and cumulative DMFT values (r_s_ = 0.382, *p* < 0.001). In contrast, no statistically significant associations were found between oral hygiene levels and missing or filled teeth in either residential group (*p* > 0.05).

## 4. Discussion

Epidemiological data in Bulgaria have consistently demonstrated an alarmingly high prevalence of dental caries among pediatric and adolescent populations. According to representative national oral health surveys conducted in 2011 and followed up in 2020, only 28.87% of children aged 5–6 years were caries-free in the primary dentition, indicating that more than 70% presented with at least one carious lesion. Oral health status deteriorated predictably with age; only 21.31% of 12-year-olds were caries-free in the permanent dentition, and this proportion declined further to 8.31% among 18-year-olds. Overall, more than 90% of adolescents under 18 years of age presented with dental caries, a level that substantially exceeds World Health Organization (WHO) oral health targets.

Mean dmft/DMFT values in Bulgaria remain elevated; among 12-year-olds, the average DMFT ranges approximately between 3.0 and 4.0, exceeding 5.0 in some areas, compared with the WHO target, which specifies a threshold below 3.0. These historical data also indicated pronounced regional and socioeconomic disparities, with consistently superior oral health indicators observed in urban areas relative to rural populations. In certain districts, caries prevalence at age 12 exceeded 90%. Furthermore, approximately two-thirds of 18-year-olds presented with gingivitis or early periodontitis, reflecting inadequate oral hygiene routines. Notably, one in four 18-year-old individuals had already experienced the extraction of at least one permanent tooth due to advanced caries complications [16].

In the present study, the mean DMFT value among 18-year-old adolescents was 7.48 ± 4.08, which stands noticeably higher than values reported in the current literature for most other nations [17,18,19]. Substantially lower DMFT metrics have been documented in Italy (3.60 among 17–19-year-olds) [20], the United States (3.25 among 16–19-year-olds) [21], Turkey (4.12 among 15–19-year-olds) [22], and Azerbaijan (3.61 among 15–19-year-olds) [23].

Closer, though still moderately lower, values have been observed in Albania (5.20) [24], while an elevated DMFT value approaching that of the present study was reported in Belarus (6.80) [25]. These cross-national differences may reflect variations in drinking water fluoridation exposure, availability of dental services, preventive strategies, and sociodemographic characteristics; however, these factors were not assessed in the present study. Nevertheless, these international comparisons should be interpreted with caution due to substantial methodological heterogeneity. The cited studies span multiple decades, employ different sampling designs, and include broader age ranges (e.g., 15–19 years) than the strictly defined 18-year-old sample in the present study. Furthermore, potential variations in visual–tactile diagnostic criteria and the lack of radiographic baseline standardization across these countries may confound direct comparisons. Consequently, greater emphasis should be placed on localized monitoring. Our results are highly consistent with contemporary Bulgarian data from comparable cohorts, such as a recent pilot study by dental researchers in Bulgaria, which documented elevated DMFT profiles among local student populations and underscored a persistent domestic public health challenge [26].

Regarding sex distribution, no statistically significant variations were observed in the cumulative DMFT index, with the notable exception of a significantly higher number of filled teeth (FT) among females. Parallel findings regarding sex-specific differences are frequent in the literature, where higher filled values or accelerated DMFT trajectories in females are often attributed to earlier permanent tooth eruption, pubertal hormonal shifts, distinct dietary preferences, and variations in salivary flow rates or biochemical compositions [27,28]. However, this trend should not be attributed solely to biological factors; it also heavily reflects documented socio-behavioral disparities in dental service utilization. Young females have traditionally demonstrated greater dental attendance, higher health-seeking behaviors, and a greater readiness to utilize restorative treatments compared to their male peers. This behavioral difference may help explain why male participants presented significantly poorer oral hygiene parameters across all OHI-S metrics (*p* < 0.05) but did not exhibit a significantly higher cumulative DMFT score. While poorer plaque control was associated with a higher number of active, untreated cavitated lesions, which may partly account for the higher raw decayed (DT) averages observed among males, the total DMFT index remains a cumulative metric of lifetime disease experience. Because the female cumulative score also reflects their higher number of restorative interventions (FT), this may partly offset the higher active decay (DT) observed among males, resulting in no statistically significant difference in the total DMFT score between the two groups.

In the present study, place of residence was significantly associated with caries experience, with higher DMFT values observed among rural participants, mainly reflecting higher mean numbers of untreated decayed and missing teeth. Interestingly, these findings contrast with certain historical investigations where an inverse urban–rural relationship was reported. Previous studies have linked urban–rural differences in caries experience to socioeconomic, behavioral, dietary, and healthcare-access factors, including differences in dietary patterns, availability of highly processed, sugar-rich foods, and geographic access to specialized dental care [29,30,31,32]. These factors may contribute to the observed rural–urban differences in caries experience.

The cohort had a mean OHI-S value of 1.89 ± 1.01. Although residential background was not significantly associated with oral hygiene levels, significant sex-based differences were observed, with males demonstrating higher OHI-S, DI-S, and CI-S scores than females. These outcomes match previous tracking data [33,34,35]. For instance, Fukai et al. noted that male cohorts frequently display poorer oral hygiene practices due to lower adherence to auxiliary hygiene measures, such as interdental flossing or antimicrobial mouthwashes, coupled with less frequent utilization of preventive dental check-ups [33].

Spearman’s correlation analysis demonstrated associations between oral hygiene status and caries experience. Positive correlations between OHI-S scores, untreated decayed teeth, and total DMFT values were present across both urban and rural populations. Previous evidence has linked differences in oral health awareness between urban and rural populations to factors such as parental education and oral health literacy [36]. However, these factors were not assessed in the present study and therefore cannot be evaluated as explanations for the observed correlations. More broadly, the contemporary public health literature also highlights the potential influence of misinformation within digital and online information environments on adolescents’ health-related motivations, which may undermine conventional preventive efforts [37].

The observed rural–urban differences may also be considered in the context of broader social determinants of health reported in previous studies. Lower household income and socioeconomic disadvantage have been associated with poorer oral health outcomes in other populations [38,39] and may contribute to geographical differences in oral health status. These findings highlight the potential relevance of socioeconomic and related social determinants when interpreting rural–urban variation in oral health.

### Limitations

This study has several limitations that warrant consideration. First, its cross-sectional design precludes the establishment of causal relationships between oral hygiene status and dental caries experience. Second, the study was conducted in a school-based sample from two Bulgarian municipalities and was not designed to provide nationally representative estimates; therefore, the findings should not be generalized to the entire population of Bulgarian 18-year-olds. School-based recruitment may also limit the external validity of the findings.

Methodologically, the clinical examinations were performed by three previously trained dentists using a standardized examination protocol, supported by a preliminary pilot study. However, formal intra- or inter-examiner reliability statistics were not calculated, and some degree of examiner-related variability cannot therefore be excluded. Furthermore, radiographic assessment was not feasible under the field conditions, which may have resulted in an underestimation of proximal and early carious lesions.

The study also did not collect detailed information on potentially important determinants of dental caries, including dietary habits, fluoride exposure, dental attendance and access to dental care, parental education, and individual or household socioeconomic status. Consequently, these factors could not be assessed as potential confounders or explanatory factors for the observed urban–rural differences. Finally, the exclusion of participants with systemic diseases may also limit the generalizability of the findings to the broader adolescent population.

## 5. Conclusions

In conclusion, dental caries experience was substantial within the examined school-based sample of 18-year-olds from these two Bulgarian municipalities. While concurrent oral hygiene levels reflect the documented OHI-S values, their weak-to-moderate correlations with the number of decayed teeth and DMFT score highlight the relationship between current oral hygiene status and cumulative caries experience across sex and residential groups. Given the cross-sectional design and the lack of representative national data, these findings cannot establish causality or determine specific socioeconomic or fluoride-related underlying factors. Nonetheless, for this specific population, the evidence supports consideration of targeted preventive and educational initiatives aimed at improving oral health among population groups with poorer oral health indicators and may inform regional oral health surveillance and preventive planning.

## Figures and Tables

**Table 1:** Mean and standard deviation of decayed, missing, filled, and mean decayed, missing, and filled teeth based on sex.

	Sex	Mean ± SD	Mean Difference (95% CI)	*t*-Test	*p*
Number of decayed teeth	male	4.22 ± 3.890	0.613 (−0.029 ÷ 1.254)	1.875	0.061
	female	3.61 ± 3.236			
Number of missing teeth	male	0.11 ± 0.435	−0.038 (−0.115 ÷ 0.040)	−0.952	0.342
	female	0.15 ± 0.429			
Number of filled teeth	male	2.99 ± 3.187	−0.883 (−1.477 ÷ −0.290)	−2.926	0.004
	female	3.88 ± 3.423			
DMFT score	male	7.32 ± 4.267	−0.308 (−1.041 ÷ 0.424)	−0.827	0.408
	female	7.63 ± 3.889			

**Table 2:** Mean and standard deviation of decayed, missing, filled, and mean decayed, missing, and filled teeth based on residence.

	Location	Mean ± SD	Mean Difference (95% CI)	*t*-Test	*p*
Number of decayed teeth	Urban	3.35 ± 3.222	−1.129 (−1.765 ÷ −0.493)	−3.488	0.001
	Rural	4.48 ± 3.844			
Number of missing teeth	Urban	0.08 ± 0.351	−0.096 (−0.173 ÷ −0.019)	−2.445	0.015
	Rural	0.18 ± 0.495			
Number of filled teeth	Urban	3.46 ± 3.459	0.058 (−0.540 ÷ 0.657)	0.192	0.848
	Rural	3.40 ± 3.209			
DMFT score	Urban	6.89 ± 4.106	−1.167 (−1.982 ÷ −0.441)	−3.161	0.002
	Rural	8.06 ± 3.980			

**Table 3:** Mean values of debris index (DI-S), calculus index (CI-S), and simplified oral hygiene index (OHI-S) stratified by sex among 18-year-old adolescents.

Indices	Sex	Mean ± SD	Mean Difference (95% CI)	*t*-Test	*p*
DI-S	male	1.74 ± 0.839	0.15 (0.011 ÷ 0.290)	2.122	0.034
	female	1.59 ± 0.710			
CI-S	male	0.28 ± 0.441	0.10 (0.033 ÷ 0.170)	2.922	0.004
	female	0.18 ± 0.309			
OHI-S	male	2.02 ± 1.122	0.25 (0.072 ÷ 0.433)	2.746	0.006
	female	1.77 ± 0.874			

**Table 4:** Assessment of simplified oral hygiene index (OHI-S) scores among 18-year-old adolescents based on place of residence.

Indices	Location	Mean ± SD	Mean Difference (95% CI)	*t*-Test	*p*
DI-S	Urban	1.64 ± 0.756	−0.05 (−0.187 ÷ 0.093)	−0.655	0.513
	Rural	1.68 ± 0.805			
CI-S	Urban	0.21 ± 0.376	−0.03 (−0.102 ÷ 0.036)	−0.950	0.342
	Rural	0.25 ± 0.391			
OHI-S	Urban	1.85 ± 0.964	−0.08 (−0.262 ÷ 0.102)	−0.865	0.387
	Rural	1.93 ± 1.059			

**Table 5:** Correlation matrix between OHI-S scores and DMFT index parameters across male and female adolescent cohorts.

		OHI-S (Male)	OHI-S (Female)
Number of decayed teeth	Spearman’s rho	0.341 **	0.252 **
	*p*	<0.001	<0.001
	*n*	240	240
Number of missing teeth	Spearman’s rho	0.055	0.044
	*p*	0.395	0.497
	*n*	240	240
Number of filled teeth	Spearman’s rho	−0.008	0.096
	*p*	0.898	0.136
	*n*	240	240
DMFT score	Spearman’s rho	0.347 **	0.307 **
	*p*	<0.001	<0.001
	*n*	240	240

** Correlation is statistically significant at the 0.01 level (2-tailed).

**Table 6:** Spearman’s correlation coefficients between simplified oral hygiene index (OHI-S) scores and DMFT components stratified by place of residence.

		OHI-S (Urban)	OHI-S (Rural)
Number of decayed teeth	Spearman’s rho	0.194 **	0.388 **
	*p*	0.003	<0.001
	*n*	240	240
Number of missing teeth	Spearman’s rho	0.041	0.035
	*p*	0.531	0.585
	*n*	240	240
Number of filled teeth	Spearman’s rho	0.116	−0.045
	*p*	0.073	0.490
	*n*	240	240
DMFT score	Spearman’s rho	0.264 **	0.382 **
	*p*	<0.001	<0.001
	*n*	240	240

** Correlation is statistically significant at the 0.01 level (2-tailed).

## Data Availability

The data presented in this study are available upon request from the corresponding author for privacy reasons.

## References

[1] World Health Organization (2024). Global Strategy and Action Plan on Oral Health 2023–2030.

[2] Glick M., Williams D.M. (2021). FDI Vision 2030: Delivering Optimal Oral Health for All. Int. Dent. J..

[3] Pitts N.B., Twetman S., Fisher J., Marsh P.D. (2021). Understanding dental caries as a non-communicable disease. Br. Dent. J..

[4] Díaz-Garrido N., Lozano C.P., Kreth J., Giacaman R.A. (2020). Competition and caries on enamel of a dual-species biofilm model with *Streptococcus mutans* and *Streptococcus sanguinis*. Appl. Environ. Microbiol..

[5] Thayumanavan T., Harish B.S., Subashkumar R., Shanmugapriya K., Karthik V. (2025). *Streptococcus mutans* biofilms in the establishment of dental caries: A review. 3 Biotech.

[6] Prabakar J., Arumugham I.M., Sri Sakthi D., Kumar R.P., Leelavathi L. (2020). Prevalence and comparison of dental caries experience among 5- to 12-year-old school children of Chandigarh using dft/DMFT and SiC Index: A cross-sectional study. J. Fam. Med. Prim. Care.

[7] Portillo-Sorto E.J. (2025). Association between dental caries and sociodemographic factors in children aged 5–12 years. Acta Odontol. Latinoam..

[8] Dăguci L., Bătăiosu M., Andrei O.C., Scrieciu M., Margaritescu C., Dascălu I., Amărăscu M., Dăguci C. (2016). Risk of dental caries for children aged 4 to 6 in Craiova. Curr. Health Sci. J..

[9] Rehman A., Ashraf H., Munir N., Syed R., Nayyar K., Tariq A. (2024). Comparative evaluation of dental caries among patients of 6–15 years age presenting to the outpatient department of Ayub College of Dentistry, Abbottabad. J. Ayub Med. Coll. Abbottabad.

[10] World Health Organization (1994). Dental Caries Levels at 12 Years.

[11] World Health Organization (2000). Global Data on Dental Caries Prevalence (DMFT) in Children Aged 12 Years.

[12] American Academy of Pediatrics (2014). Teen Stages of Adolescence. Healthy Children: Ages & Stages. https://www.healthychildren.org/English/ages-stages/teen/Pages/Stages-of-Adolescence.aspx.

[13] Grace T.W. (1998). Health problems of late adolescence. Prim. Care.

[14] World Health Organization (1997). PAHO/WHO Oral Health Survey: Basic Methods.

[15] Greene J.C., Vermillion J.R. (1964). The simplified oral hygiene index. J. Am. Dent. Assoc..

[16] Ministry of Health of the Republic of Bulgaria Draft National Program for the Prevention of Oral Diseases in Children from 0 to 18 Years in the Republic of Bulgaria, 2026–2030. https://www.mh.government.bg/bg/normativni-aktove/proekti-na-normativni-aktove/107.

[17] Žemaitienė M., Grigalauskienė R., Vasiliauskienė I., Saldūnaitė K., Razmienė J., Slabšinskienė E. (2016). Prevalence and severity of dental caries among 18-year-old Lithuanian adolescents. Medicina.

[18] Yavnai N., Mazor S., Vered Y., Shavit I., Zini A. (2020). Caries prevalence among 18-year-olds: An epidemiological survey in Israel. Isr. J. Health Policy Res..

[19] Johanson V., Söderfeldt B., Axtelius B. (2007). Oral B’s Nordic Report on Oral Health.

[20] Arcella D., Ottolenghi L., Polimeni A., Leclercq C. (2002). The relationship between frequency of carbohydrate intake and dental caries: A cross-sectional study in Italian teenagers. Public Health Nutr..

[21] Beltrán-Aguilar E.D., Barker L.K., Canto M.T., Dye B.A., Gooch B.F., Griffin S.O., Hyman J., Jaramillo F., Kingman A., Nowjack-Raymer R. (2005). Surveillance for dental caries, dental sealants, tooth retention, edentulism, and enamel fluorosis—United States, 1988–1994 and 1999–2002. MMWR Surveill. Summ..

[22] Kargul B., Bakkal M. Systems for the Provision of Oral Health Care in the Black Sea Countries. Part 6: Turkey. OHDMBSC 2010, 9. https://cecdo.org/wp-content/uploads/2014/09/Oral-Health-Turkey-Sept-2010.pdf.

[23] Pashayev A.C., Mammadov F.U., Huseinova S.T. An Investigation into the Prevalence of Dental Caries and Its Treatment Among the Adult Population with Low Socioeconomic Status in Baku, Azerbaijan. OHDM 2011, 10. https://www.walshmedicalmedia.com/open-access/an-investigation-into-the-prevalence-of-dental-caries-and-its-treatment-among-the-adult-population-2247-2452-10-431.pdf.

[24] Laganà G., Abazi Y., Beshiri Nastasi E., Vinjolli F., Fabi F., Divizia M., Cozza P. (2015). Oral health conditions in an Albanian adolescent population: An epidemiological study. BMC Oral Health.

[25] Shadiev K.K., Leous A.P., Pakhomov G.N. (1988). Significance of the WHO Criteria in National Achievement of Oral Health Objectives.

[26] Levterova B., Tomova Z., Tomov D., Uzunova Y. (2026). Oral Health Status (DMFT Index) and Hygiene Practices Among Dental Students in Bulgaria: A Pilot Study. Dent. J..

[27] Lukacs J.R., Largaespada L.L. (2006). Explaining sex differences in dental caries prevalence: Saliva, hormones, and life-history etiologies. Am. J. Hum. Biol..

[28] Dodds M.W., Johnson D.A., Yeh C.K. (2005). Health benefits of saliva: A review. J. Dent..

[29] Mielnik-Błaszczak M., Rudnicka-Siwek K., Warsz M., Struska A., Krajewska-Kurzępa A., Pels E. (2016). Evaluation of oral cavity condition regarding decay in 18-year-olds from urban and rural areas in Podkarpackie Province, Poland. Przegl. Epidemiol..

[30] Mandal K.P., Tewari A.B., Chawla H.S., Gauba K.D. (2001). Prevalence and severity of dental caries and treatment needs among populations in the eastern states of India. J. Indian Soc. Pedod. Prev. Dent..

[31] Irigoyen M.E., Luengas I.F., Yashine A., Mejía A.M., Maupomé G. (2000). Dental caries experience in Mexican schoolchildren from rural and urban communities. Int. Dent. J..

[32] Kalita C., Choudhary B., Saikia A.K., Sarma P.C. (2016). Caries prevalence of school-going boys and girls according to cleaning methods and soft drink-taking frequency in and around Guwahati City. J. Indian Soc. Pedod. Prev. Dent..

[33] Hamasha A.A., Alshehri A., Alshubaiki A., Alssafi F., Alamam H., Alshunaiber R. (2018). Gender-specific oral health beliefs and behaviors among adult patients attending King Abdulaziz Medical City in Riyadh. Saudi Dent. J..

[34] Al-Ansari J., Honkala E., Honkala S. (2003). Oral health knowledge and behavior among male health sciences college students in Kuwait. BMC Oral Health.

[35] Shiau H.J., Reynolds M.A. (2010). Sex differences in destructive periodontal disease: A systematic review. J. Periodontol..

[36] Doley S., Srivastava M., Gupta R., Piplani A. (2022). Association between oral hygiene status and dental caries among 13–14-year-old children of Kamrup District, Assam. J. Indian Assoc. Public Health Dent..

[37] Alhomsi A., Aldoss H., Aljoujou A.A., Mashlah A.M., Hajeer M.Y., Alyafi A., Almasri I.A. (2024). Exploring How People Interact With Dental Misinformation on Social Media: A Cross-Sectional Study. Cureus.

[38] World Health Organization (2010). A Conceptual Framework for Action on the Social Determinants of Health.

[39] Habal W., Alkattan R., Hajeer M.Y., Alkhouli M., Al-Nerabieah Z., Habal T., Awawdeh M. (2024). Impact of Syrian Conflict on the Oral Health of Adolescents: A Cross-Sectional Study. Cureus.

